# Proteomic insights into the invasiveness and tumor progression of non‐functioning pituitary adenomas: A scoping review

**DOI:** 10.1111/jne.70148

**Published:** 2026-03-07

**Authors:** Thomas Skoglund, Linus Köster, Annika Thorsell, Oskar Ragnarsson, Gudmundur Johannsson, Tobias Hallén

**Affiliations:** ^1^ Department of Neurosurgery Sahlgrenska University Hospital Gothenburg Sweden; ^2^ Department of Clinical Neuroscience Institute of Neuroscience and Physiology, Sahlgrenska Academy, University of Gothenburg Gothenburg Sweden; ^3^ Proteomics Core Facility at Sahlgrenska Academy Gothenburg University Gothenburg Sweden; ^4^ Department of Medicine Sahlgrenska University Hospital Gothenburg Sweden; ^5^ Department of Internal Medicine and Clinical Nutrition Institute of Medicine, Sahlgrenska Academy, University of Gothenburg Gothenburg Sweden; ^6^ Wallenberg Center for Molecular and Translational Medicine, University of Gothenburg Gothenburg Sweden

**Keywords:** invasiveness, mass spectrometry, nonfunctioning pituitary adenoma, quantitative proteomics, tumor progression

## Abstract

Nonfunctioning pituitary adenomas (NFPAs) are common intracranial tumors that, despite being histologically benign, can exhibit invasive growth, as well as postoperative tumor progression. Surgical resection is the primary treatment of choice; however, residual tumor tissue is frequently observed, with between 30% and 50% of these cases subsequently experiencing regrowth. The molecular mechanisms governing NFPA behavior remain poorly understood, and robust prognostic biomarkers are still lacking despite genomic and transcriptomic studies. Mass spectrometry (MS)‐based proteomics enables large‐scale, global protein quantification and monitoring of changes in protein expression, which could identify markers of tumor behavior as well as potential new therapeutic targets. This review synthesizes existing proteomic research on NFPAs and identifies candidate biomarkers and dysregulated pathways associated with invasiveness and tumor progression. We used PubMed, the Cochrane Library, and Scopus to perform a structured and comprehensive literature search of studies published since the year 2000 that applied MS‐based proteomics to evaluate NFPAs. The identified studies were grouped into three main categories: (1) proteomic differences between NFPAs and normal pituitary glands, (2) biomarkers linked with tumor progression, and (3) molecular signatures distinguishing invasive from noninvasive NFPAs. Among the 30 included studies, 15 compared NFPAs with normal pituitary tissue and reported altered protein expression, metabolic reprogramming, and spliceosome dysregulation. Only two studies addressed tumor progression, showing associations with RNA processing, energy metabolism, and β‐catenin phosphorylation. Studies evaluating NFPA invasiveness (*n* = 16) highlighted altered extracellular matrix remodeling and dysregulated PI3K–Akt and MAPK/ERK signaling along with specific proteins, including Ezrin and β‐catenin. Across themes, recurrent alterations in MAPK/ERK, PI3K–Akt–mTOR, Wnt/β‐catenin, and IL6/JAK/STAT3 signaling suggest that NFPA biology is driven by interconnected pathways rather than isolated molecular events. Sample sizes were generally small, with more than 50% of studies analyzing less than 10 NFPAs, and only one study including up to 100 NFPAs. Methodological heterogeneity and lack of validation remain major limitations. Although modern proteomic studies provide valuable insights into NFPA biology and particularly invasiveness, investigations on mechanisms of progression are limited. Moreover, robust biomarkers have not yet been established, and most findings remain exploratory due to small sample sizes and methodological heterogeneity. Future research should focus on larger, prospective cohorts, integration of clinical and imaging data with multi‐omics approaches, and standardized protocols for sample handling and preparation to enhance reproducibility. Such efforts are needed to translate proteomic discoveries into clinically useful biomarkers and novel therapeutic strategies.

## 1 INTRODUCTION

Nonfunctioning pituitary adenomas (NFPAs), also entitled nonfunctioning pituitary neuroendocrine tumors according to the current World Health Organization (WHO) classification, represent a significant proportion of pituitary tumors encountered in clinical practice. Although functioning adenomas are often diagnosed early due to hormone‐related symptoms, NFPAs are usually detected after growing large enough to cause mass effects, such as visual impairment and hypopituitarism.^1^ Another concern is their capacity for invasive growth, which poses significant challenges to surgical management. This growth can lead to infiltration of surrounding structures, such as the cavernous sinus, making complete resection difficult.^2^ First‐line treatment involves surgery, preferably via the transsphenoidal approach; however, residual tumor tissue is frequently observed after surgery, and although NFPAs are nearly always histologically benign, their behavior varies widely. Invasive growth patterns are associated with higher rates of postsurgery tumor progression and increased mortality rates.^3^ The most recent WHO classification of pituitary tumors emphasizes transcription factor profiling as a means of defining tumor lineage and providing a more accurate diagnostic framework for differentiating subtypes.^4^ However, this molecular classification rarely aids in predicting tumor invasiveness or risk of progression, both of which remain pressing clinical challenges.

Despite insights from genomic and transcriptomic studies, the underlying mechanisms of invasiveness and progression, as well as the factors driving these activities, remain poorly understood.^5^ This underscores the critical need for reliable prognostic markers to aid prediction of clinical courses and guide tailored therapeutic strategies for residual and invasive tumors.

Proteomics is the global study of proteins expressed by a cell, tissue, or organism at a specific point in time. Thousands of proteins can be quantified, identifying protein‐expression patterns, post‐translational modifications (PTMs), and pathway alterations, offering valuable biomarkers and therapeutic targets across diseases and particularly in cancers.^6^

Recent advances in mass spectrometry (MS)‐based clinical proteomics, including automated sample preparation and high resolution/high mass accuracy data‐independent acquisition (DIA) strategies, have markedly improved proteome coverage, reproducibility, and analytical throughput. These developments now enable the study of larger patient cohorts and are accelerating the transition of proteomics into applications that directly support clinical practice, positioning it as a key component of precision medicine.

Proteomic approaches offer the potential to uncover biomarkers or protein panels and molecular pathways relevant to understanding pituitary adenoma behavior. However, no synthesis of proteomic studies focusing specifically on NFPAs has been published. This scoping review aims to clarify current knowledge, pointing at putative candidate biomarkers and molecular mechanisms linked to tumor progression and invasiveness, and summarize methodological limitations that may inform future translational research.

## 2 MATERIALS AND METHODS

### 2.1 Search strategies

To ensure comprehensive coverage of relevant studies, we conducted searches using a variety of terms related to proteomics and NFPAs. These searches were performed across the Cochrane Library, PubMed, and Scopus databases and targeted English‐language studies. The specific search strings employed are provided in Table S1. The initial searches were conducted at the Central Clinical Library of Sahlgrenska University Hospital, Gothenburg, Sweden, on November 21, 2024, and updated on August 29, 2025.

**FIGURE 1:**
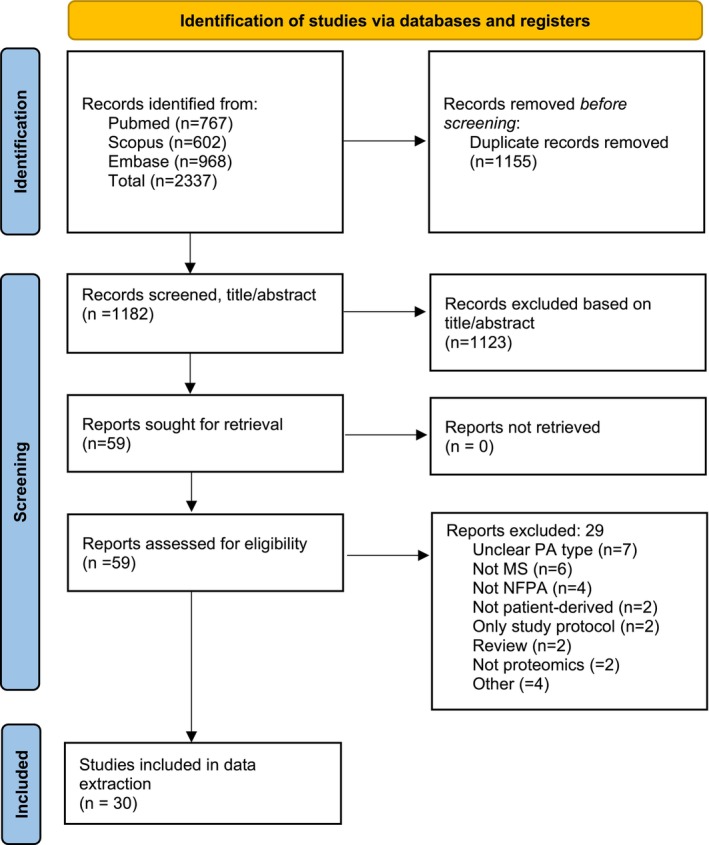
Flow chart describing the criteria for study inclusion.

After gathering the records, duplicate citations were removed using a structured de‐duplication approach in EndNote, and two reviewers (TH and TS) independently screened titles and abstracts. Disagreements were resolved through discussion. Inclusion criteria targeted peer‐reviewed, original studies with full‐text availability and examining proteomic analyses of NFPAs in patients who had undergone surgical intervention. Studies were included if they reported on the use of MS techniques and were published in the year 2000 or later. We chose the year 2000 as the cut‐off to capture the advent of electrospray ionization Liquid Chromatography–Mass Spectrometry (LCMS) shotgun proteomics and to reflect the transition from traditional two‐dimensional gel electrophoresis (2D‐GE) and gel‐based quantification to LCMS‐based quantification strategies. This timeframe also captures the development of high resolution/high mass accuracy Orbitrap technology, which revolutionized the proteomics field and greatly enhanced the depth and precision of proteomic analyses. Exclusion criteria included studies focusing exclusively on functioning pituitary adenomas, grey literature, non‐English‐language publications, and review articles. Studies including both functioning and nonfunctioning adenomas were also excluded if results for NFPAs were not reported separately. The process of article selection followed the PRISMA extension for scoping reviews (PRISMA‐ScR) and is described in Figure 1.^7^

As this review was conducted as a scoping review, no formal risk‐of‐bias or methodological quality assessment of individual studies was performed. No review protocol was registered prior to study initiation.

### 2.2 Data extraction

Data extraction was conducted by three reviewers (LK, TH, and TS), with each article independently assessed by at least two. Any data‐specific questions or uncertainties were resolved by consensus among all three reviewers. For each included study, we extracted and tabulated information on the primary research objective, patient cohort and sample size, proteomic methodology, additional ‐omics techniques, analytical tools, reported clinical correlations, and key proteomic findings. The extracted data were jointly reviewed and validated by all team members. As nomenclature was inconsistently reported across studies, protein names were listed first, followed by the corresponding italicized gene symbols in parentheses, standardized according to UniProt nomenclature (www.uniprot.org). When protein names were used in the original articles, these were retained unchanged.

## 3 RESULTS

**FIGURE 2:**
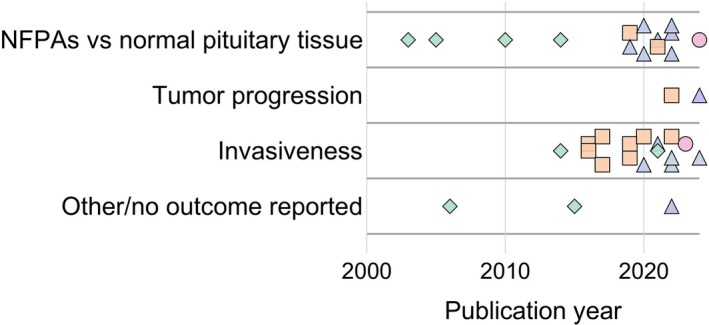
Distribution of studies on proteomic analyses of nonfunctioning pituitary adenomas (NFPAs) by publication year and reported outcomes. Categories include comparisons to normal pituitary tissue, tumor progression, invasiveness, and studies with other or no specific outcomes reported. Studies with multiple foci or outcomes are represented by markers corresponding to each category. A temporal shift in the proteomic techniques applied across publication years can be observed. The symbols denote the proteomic techniques used: (♦) gel‐based proteomics, (■) isobaric labeling quantitative proteomics, (▲) label‐free DDA, and (●) label‐free DIA.

The database search yielded 1182 unique records, of which 30 were selected based on their focus on proteomic analyses related to NFPAs.^8^, ^9^, ^10^, ^11^, ^12^, ^13^, ^14^, ^15^, ^16^, ^17^, ^18^, ^19^, ^20^, ^21^, ^22^, ^23^, ^24^, ^25^, ^26^, ^27^, ^28^, ^29^, ^30^, ^31^, ^32^, ^33^, ^34^, ^35^, ^36^, ^37^ Most of these studies could be categorized into three thematic areas: (i) proteomic differences between NFPAs and normal pituitary glands (*n* = 15), (ii) proteomic profiles associated with tumor progression (*n* = 2), and (iii) proteomic markers distinguishing invasive and noninvasive NFPAs (*n* = 16). Studies addressing more than one thematic area were included under multiple headings to reflect their contributions to different aspects of NFPA biology. Throughout the Results section, brief descriptors of the underlying studies are used selectively to provide context. Detailed study characteristics are summarized in Table 1, which describes patient cohorts, sample sizes, proteomic techniques, analytical tools, and key findings. Figure 2 shows the distribution of publication years.

**TABLE 1:** Overview of included studies on MS‐based proteomic analyses of nonfunctioning pituitary adenomas. All abbreviations used in this table are defined in Supplementary Table S2.

Paper	Primary research objective	Patient cohort and sample size (proteomics)	Proteomic analysis techniques^a^	Other techniques used or deposited data sets	Tools and database for protein identification and quantification	Tools for functional and pathway analysis	Clinical relevance reported	Proteomic results reported	Key findings
Zhan et al.^8^	Identify and characterize DEPs in NFPAs, with a focus on secretagogin, to understand its role in tumorigenesis	15 NFPA samples and 8 normal pituitary tissues	2D‐GE, MALDI‐TOF MS and LC‐QIT MS/MS	Transcriptomics	PDQuest, SWISS‐PROT/TrEMBL, NCBI, Mascot, MS‐FIT, SEQUEST	EMBL	No clinical outcomes; comparisons made with normal pituitary tissue	Secretagogin significantly downregulated in NFPAs compared to normal pituitary tissue	Secretagogin significantly downregulated in NFPAs, may play a role in NFPA patophysiology
Moreno et al.^9^	Classify NFPAs by gene expression and proteomic profiles to uncover pathways	11 NFPA and 8 normal pituitary samples	2D‐GE, MALDI‐TOF MS and LC‐QIT MS/MS	Transcriptomics with gene expression validation	PDQuest, SWISS‐PROT/TrEMBL, Mascot, Peptident, Sequest	KEGG, DAVID	No clinical outcomes; comparisons made with normal pituitary tissue	50 DEPs identified in NFPAs compared to normal pituitary tissue	Altered expression of SFRP1, TLE2, PITX2, NOTCH3, and DLK1, indicates activation of the Wnt and Notch developmental pathways, potentially driving NFPA progression
Zhan et al.^10^	Characterize endogenous nitroproteins and their interacting proteins in a NFPA	Pituitary adenoma from 1 patient (null‐cell PA)	Nitroprotein enrichment, MALDI–LTQ MS/MS	N/A	PDQuest, SWISS‐PROT, NCBI	ScanProsite, MotifScan	None reported	9 nitroproteins linked to tumorigenesis identified	First mapping of nitroproteins in NFPAs; protein nitration may play a key role in NFPA formation
Zhan et al.^11^	Define key signaling pathway networks involved in pituitary adenoma pathogenesis	11 NFPAs, 4 PRLs and 8 normal pituitary tissues used for comparative proteomics + pathway analysis of available proteomic data	2D‐GE, MALDI‐TOF MS and LC‐QIT MS/MS	N/A	PDQuest, SWISS‐PROT	IPA‐KB, ExPASy	No clinical outcomes; comparisons with normal pituitary tissue	111 proteins identified, 56 DEPs, 17 nitroproteins	Mitochondrial dysfunction, oxidative stress, cell‐cycle dysregulation, and MAPK signaling identified as key pathways in pituitary adenomas, suggesting biomarker and therapeutic targets
Zhan et al.^12^	Discover molecular markers and pathway networks associated with tumor invasiveness	8 NFPA samples (4 invasive, 4 noninvasive)	2D‐GE, MALDI‐TOF‐MS and LC‐qTOF‐MS/MS	N/A	PDQuest, SWISS‐PROT, Mascot	GO (via DAVID), IPA	Tumor invasiveness	57 DEPs identified; key pathways: mitochondrial dysfunction, oxidative stress, MAPK signaling, TR/RXR activation	Altered pathway networks specific to invasive NFPAs; GRP78 and CDK5 proposed as novel biomarkers and therapeutic targets for managing invasiveness
Zhan et al.^13^	Investigate proteome heterogeneity among NFPA subtypes to identify subtype‐specific mechanisms and biomarkers	12 NFPA tissues and 8 normal pituitary tissues	2D‐GE, MALDI‐TOF MS and LC‐QIT MS/MS	N/A	PDQuest, UniProt, NCBI, sequest	IPA‐KB	Subtypes of NFPA and compared to normal pituitary tissue	44 DEPs common to all NFPA subtypes	Subtype‐specific proteomic profiles identified in NFPA; 4 key pathways: MAPK‐signaling, oxidative stress, mitochondrial dysfunction, and cell‐cycle dysregulation
Wang et al.^14^	Characterize the proteomic and functional profiles of follicle‐stimulating hormone (FSH)‐positive NFPAs	One FSH‐positive NFPA sample	2D‐GE with MALDI‐TOF MS	N/A	PDQuest, SWISS‐PROT, Mascot	DAVID (GO), IPA‐KB	None reported	107 nonredundant proteins detected	Pathway analysis identified gluconeogenesis, glycolysis, mitochondrial dysfunction, oxidative stress, cell cycle regulation, MAPK signaling, immune response, TP53 signaling, VEGF signaling, and inflammatory pathways but no comparison was performed
Feng et al.^15^	Investigate molecular expression patterns and key signaling pathways driving pituitary null cell adenoma invasion	5 invasive and 4 noninvasive null cell PAs	iTRAQ 2‐plex pooled groups, LC‐TripleTOF MS/MS	Transcriptomics	ProteinPilot, SWISS‐PROT, Mascot	GO, IPA‐KB	Tumor invasiveness.	4568 proteins identified; 283 DEPs. Key pathway linked to invasiveness: IL‐6R/JAK2/STAT3/MMP9 signaling	IL‐6R/JAK2/STAT3/MMP9 pathway overactivation drives PNCA invasion. DEPs enriched in cell migration and acute phase response signaling pathways. IL‐6 identified as a critical upstream regulator
Yu et al.^16^	Study molecular changes between invasive and noninvasive NFPAs	5 invasive and 4 noninvasive NFPAs	iTRAQ, LC‐TripleTOF MS/MS	Transcriptomics	ProteinPilot, SWISS‐PROT, Mascot	IPA‐KB	Tumor invasiveness	433 DEPs between invasive and noninvasive NFPAs. 29 molecules were enriched in 25 canonical signaling pathways	8 invasion‐associated genes identified; decreased *CHGA* and increased CLU expression validated as potential invasion‐related therapeutic targets
Chen et al.^17^	Identify biomarkers associated with invasiveness in the three subtypes of NFPAs (null cell adenomas, oncocytomas, and gonadotroph adenomas)	5 invasive NFPAs and 4 noninvasive NFPAs used for proteomic analyses	iTRAQ, LC‐TripleTOF MS/MS	Western blot, transcriptomics	ProteinPilot, SWISS‐PROT, Mascot	IPA‐KB	Tumor invasiveness	3568 proteins identified; 421 DEPs in invasive versus noninvasive NFPAs	Ezrin identified as a key invasive biomarker in NFPAs; higher expression in invasive subtypes correlates with increased invasiveness; functional studies confirm Ezrin's role in promoting cell invasion. Inhibition of Ezrin expression possible target
Zhang et al.^18^	Investigate the role of fibroblasts in bone destruction	4 bone destructing NFPAs and 4 not bone destructing NFPAs	Enrichment of low abundant proteins with ProteoMiner protein enrichment, TMT 2‐plex pooled groups, LC‐LTQ MS/MS	IHC, Cytokine arrays	Proteome Discoverer, UniProt/SWISS‐PROT	Gene Cluster, Cytoscape, STRING, GO, KEGG, DAVID	Tumor invasiveness (bone destruction)	895 proteins in NBD‐NFPA fibroblasts and 747 proteins in BD‐NFPA fibroblasts, with 497 proteins overlapping	Increased osteopontin and caldesmon expression in BD‐NFPA fibroblasts correlates with bone destruction; enriched pathways include cytoskeleton regulation and Actin remodeling
Cheng et al.^19^	Proteomic profiling of NFPAs to explore molecular mechanisms and discover potential biomarker	6 NFPA tissue samples and 4 pituitary gland tissues (WB)	TMT 6‐plex, LC‐OrbitrapFusion MS/MS	Western blot, transcriptomics on deposited data sets	Proteome Discoverer, UniProt/SWISS‐PROT, Mascot	GO, KEGG, PANTHER, Cytoscape, STRING, DAVID	None reported	6076 proteins identified; 1088 overlapped molecules (proteins and DEGs) from proteomic and transcriptomic data; key pathways: endocytosis, spliceosome, focal adhesion, and platelet activation	Significant molecular pathways and hub molecules (e.g., SRC, FAK, AKT1, ACACA) related to tumor behavior; key proteins involved in cell adhesion and metabolic processes validated
Feng et al.^20^	Characterize metabolic alterations in pituitary adenoma subtypes using multiomic approaches	56 cases (10 corticotroph, 10 gonadotroph, 8 somatotroph, 8 mammosomatotroph, 5 lactotroph, 10 oncocytomas, 5 null cell adenomas) and 7 normal pituitary tissues	iTRAQ 6‐plex pooled groups, LC‐TripleTOF MS/MS	Metabolomics, transcriptomics	ProteinPilot, SWISS‐PROT, Mascot	None reported	Comparing subtypes of PAs and normal pituitary tissue	Integrated with metabolomics, proteomic analysis highlighted altered glucose metabolism pathways, with IDH2 identified as a key regulator in GH‐PA	Pituitary adenomas showed reduced glucose metabolism, including both glycolysis and glucose oxidation; *IDH2* identified as a potential target for intervention in GH‐PA
Long et al.^21^	To construct and validate integrative molecular networks based on multi‐omics data to understand the molecular mechanisms of NFPAs	4 NFPAs and 4 control pituitary tissues	PTMscan enrichment of phosphopeptides, LFQ, LC‐QExative Orbitrap MS/MS	Western blot, Proteomics and transcriptomics on deposited datasets	UniProt/SWISS‐PROT, NCBI, sequest, Progenesis, Skyline	IPA‐KB	None reported for the tissue samples analyzed but tumor invasiveness was used as outcome in omic data from public databases	1006 unique phosphorylated‐sites within 409 proteins identified, more than 19 pathways involved. 19 high‐frequency hub‐molecules validated in NFPAs with PTMScan; key pathways: PI3K/AKT, mTOR, Wnt, ERK/MAPK signaling	Multi‐omics analysis identified 62 molecular networks and 519 canonical pathways; key pathways (e.g., ERK/MAPK, PI3K/AKT, mTOR, Wnt) significantly altered in NFPAs, highlighting potential biomarkers and therapeutic targets
Wang et al.^22^	Identify molecular characteristics of FSH‐positive NFPAs	6 NFPAs (3 FSH‐positive and 3 FSH‐negative)	TMT 6‐plex, LC‐OrbitrapFusion MS/MS	Western blot, transcriptomics on deposited datasets	Proteome Discoverer, SWISS‐PROT, Mascot	GO, KEGG, STRING, Cytoscape, KOBAS	None reported (tumor invasiveness in database material)	594 DEPs identified between FSH‐positive and FSH‐negative NFPAs	Three significant pathways associated with tumor invasiveness: ECM‐receptor interaction, focal adhesion, and PI3K‐Akt signaling, significantly upregulated DEPs (*ITGA1*, *ITGA6*, and *ITGB4*)
Eieland et al.^23^	Investigate signaling pathways underlying differences in hormone production, secretion, growth, and aggressiveness between functioning (FCA) and silent (SCA) corticotroph adenomas	12 FCA and 12 SCA	LFQ, LC‐Q Exactive Orbitrap MS/MS	Transcriptomics	MaxQuant, UniProt	Perseus, STRING, PANTHER	Silent vs. functional corticotroph adenoma	2782 proteins identified; 170 DEPs. Significantly different expression, in particular POMC, TBX19, and PSCK1	Distinct signaling pathways in corticotroph adenomas: increased protein processing in FCA and adhesion molecules linked to SCA aggressiveness
Liu et al.^24^	Integrate phosphoproteomics and transcriptomics to identify molecular mechanisms of invasiveness	4 PAs and 4 normal pituitary tissues	TiO2 enrichment of phosphopeptides, TMT 6‐plex pooled sample, LC‐Q exactive Orbitrap MS/MS	Transcriptomics on deposited datasets	UniProt, NCBI, Mascot	Cytoscape, DAVID, STRING, KEGG	Tumor invasiveness and comparison to normal pituitary tissue	1035 phosphoproteins with 2982 phosphorylation sites identified in NFPAs vs. Controls; 2751 DEGs were identified in invasive NFPAs versus controls	12 statistically significant signaling pathways associated with tumor invasiveness identified
Taniguchi‐Ponciano et al.^25^	Investigate the role of the spliceosome in PAs using proteomic and transcriptomic	14 PAs (8 NFPA, 4 GH‐secreting, 1 TSH‐secreting, 1 prolactinoma), 6 normal pituitary tissues	LC qTOF MS/MS	Transcriptomics, IHC	Protein Scape, Mascot, SWISS‐PROT for identification only	GEO (NCBI)	Subtypes of PAs and compared to normal pituitary tissue	3060 DEPs identified in NFPA samples	Spliceosome components and splicing profiles serve as markers and potential therapeutic targets for PA subtypes
Carrillo et al.^26^	Analyze proteomic profiles of invasive and noninvasive PAs	53 NFPAs (49 invasive, 4 noninvasive) and 11 functioning PAs (7 invasive, 4 noninvasive)	2D‐GE, LC‐LTQ MS/MS	N/A	Bio1D EvolutionCapt, Proteome Discoverer, SEQUEST	None reported	Tumor invasiveness	11 DEPs identified; key functions: metabolic enzymes, cellular signals, cell structure, and energy metabolism	Hint1 overexpression linked to invasiveness in PAs; proposed as a potential biomarker and therapeutic target
Li et al.^27^	Investigate phosphorylation‐mediated pathway alterations in NFPAs	4 pooled NFPAs and 4 pooled samples from normal pituitary tissues	TiO_2_ enrichment of phosphopeptides, TMT on pooled groups, LC‐Q Exactive Orbitrap MS/MS	N/A	MaxQuant, UniProt	KEGG, DAVID, GO	PA vs. normal pituitary tissue	595 differentially phosphorylated proteins (DPPs) with 1412 phosphosites identified compared to controls; 9 significant pathways	Key pathways include spliceosome, RNA transport, and proteoglycans in cancer; phosphorylation of calnexin contributes to ER‐processing (endoplasmatic reticulum) dysfunction
Wen et al.^28^	Investigate protein acetylation profile alterations and their roles in NFPA pathways	4 NFPAs and 4 normal pituitary tissues	Enrichment of acetylated peptides, LFQ LC‐Q exactive Orbitrap MS/MS	Western blot, transcriptomics on deposited datasets	MaxQuant	GO, KEGG, DAVID, Motif‐X, GEO	Comparison between NFPAs and normal pituitary tissue (tumor invasiveness was used as outcome in omic data from public databases)	296 acetylated proteins with 517 acetylation sites identified, 76 differentially acetylated sites with the majority showing significant down‐acetylation in NFPAs	Acetylation‐mediated molecular pathway changes in NFPAs linked to metabolic reprogramming, translation, cell adhesion, and oxidative stress
Rai et al.^29^	Characterize the phosphorylation patterns of noninvasive, invasive, and recurrent NFPAs	11 gonadotroph adenomas (SF1‐lineage), 2 corticotroph tumors (TPIT‐lineage), 3 immature PIT1‐lineage (2 GH +ve, 1 PRL +ve) and 4 null cell tumors	TiO_2_ enrichment of phosphopeptides, TMTon pooled groups, LC‐OrbitrapFusion or Velos MS/MS	IHC, Western Blot	Proteome Discoverer, NCBI Human RefSeq 70, SEQUEST, Mascot	FunRich	Noninvasive, invasive, and recurrent disease groups compared	3185 quantified phosphopeptides identified; 566 altered in invasive group, 1113 altered in recurrent group	Cluster of 22 upregulated phosphopeptides in recurrent NFPAs; β‐catenin Ser552 phosphorylation (Wnt/β‐catenin pathway) proposed as a biomarker for NFPA recurrence
Ren et al.^30^	Investigate exosomal protein and mRNA content and its role in NFPA tumorigenesis	9 invasive and 12 noninvasive NFPAs +6 prolactinomas, 6 GH‐secreting, 6 ACTH‐secreting adenomas, and 6 normal pituitary tissues	QExative Orbitrap MS/MS	Western blot, transcriptomics	ProteoWizard, SWISS‐PROT for identification only, Mascot, Scaffold	IPA	Tumor invasiveness	1506 proteins identified in invasive NFPAs, 217 proteins in noninvasive NFPAs	Increased expression of matrix metalloproteinase‐1 (MMP1) and its formation in exosomes (exo‐MMP1) correlated with NFPA invasiveness
Taniguchi‐Ponciano et al.^31^	Examine expression profiles of kinases, cyclins, and CDKs in pituitary adenomas	POU1F1 adenomas (GH‐, TSH‐, and PRL‐, n = 16), NR5A1 (gonadotropes and null cell, *n* = 17), TBX19 (ACTH‐, *n* = 6), silent ACTH‐tumors (*n* = 3) and 6 normal pituitary tissues	LC‐qTOF MS/MS	Transcriptomics, IHC	Protein Scape, SWISS‐PROT, Mascot for identification only	Enrichr, Metascape	No clinical outcome reported. Three different adenoma lineages compared	NR5A1‐, POU1F1‐, and TBX19‐derived tumors showed distinct kinase expression, with subtype‐specific upregulation and alterations in MAPK, insulin, VEGFA, and phospholipase D signaling	Distinctive proteomic profiles for kinases for each of the 3 PA lineages
Zhang et al.^32^	Identify diagnostic markers, refine classification, and explore therapeutic targets for NFPAs through integrative multi‐omics analysis	200 PAs (100 NFPAs) + independent cohort validation (750 PAs) and 7 normal pituitary tissues	FeNTA enrichment of phosphopeptides, LC‐Orbitrapf FusionLumos MS/MS	Genomics, transcriptomics, IHC	Proteome Discoverer, Mascot, Human RefSeq, Firmiana	GSEA, NetworKIN	Tumor invasiveness and subtype‐specific molecular features.	10,011 proteins and 29,219 phosphosites identified; 7 predictive of PAs identified	EMT markers (e.g., *FN1*) and ECM‐remodeling enzymes (e.g., MMP8) enriched in invasive clusters; multi‐omics clustering revealed seven subtypes, aiding in novel classification and potential therapeutic targets.
Zhao et al.^37^	Investigate the role of ubiquitination in regulating ACTH secretion in silent corticotroph adenomas (SCAs)	5 SCA samples and 5 functioning corticotroph adenomas (FCA)	PTM enrichment, LFQ LC‐TimsTOF MS/MS	None	MaxQuant, SWISS‐PROT	GO, KEGG, STRING, GSEA, UbiBrowser, Motif‐X	Secreting versus nonsecreting corticotroph adenoma	111 ubiquitinated sites identified on 94 proteins; 102 sites downregulated in SCAs; key pathways: vesicle and protein secretion processes	Decreased ubiquitination of ATP7A identified as a regulator of ACTH secretion in SCAs; potential therapeutic target suggested
Zhang et al.^33^	Identify a novel invasion biomarker associated with epithelial‐mesenchymal transition (EMT) in PAs	19 PAs (10 invasive PA SCA and 9 non‐invasive PA FSH)	LFQ DIA LC‐Q exacative Orbitrap HF MS/MS	Genomics, transcriptomics	Uniprot, MaxQuan library generation, Spectronaut	GO, KEGG, GeneCards, STRING, Cytoscape, GeneMANIA	Tumor invasiveness.	5598 proteins identified. 833 DEPs; 46 EMT‐related DEPs, including 6 overlapping hub/core EMT‐DEPs	*SLC2A1* is significantly upregulated in invasive PAs; potential predictive marker involved in EMT regulation
Candy et al.^34^	Catalog differential membrane protein expression in NFPA compared to pituitary glands	20 NFPA samples (mixed subtypes) and 10 normal pituitary tissues	LFQ LC‐Qexactive Orbitrap MS/MS, DDA and DIA	N/A	Spectronaut	GO, Reactome, clusterProfiler	None reported	6492 proteins identified; 2110 DEPs between adenoma samples and normal pituitary tissue	Membrane receptors NOTCH3 and PTPRJ upregulated in NFPA compared to normal pituitary tissue
Hallén et al.^35^	Identify proteomic profiles associated with postoperative tumor progression	46 NFPAs (gonadotroph); 29 with post‐surgical tumor progression and 17 stable	LFQ LC‐Qexactive Orbitrap MS/MS	N/A	Proteome Discoverer; UniProt, SWISS‐PROT, Mascot	GO, STRING, Reactome, Perseus, enrichPathway	Postoperative tumor progression of residual tumor.	4074 proteins were identified. 550 DEPs identified, linked to pathways involved in tumor aggressiveness	Distinct proteomic profiles between stable and progressive tumors, *SNRPD1*, SRSF10, SWAP‐70, *PSMB1* associated with progression; functional enrichment analysis indicated processes involving translation, ROBO‐receptor signaling, energy metabolism, mRNA metabolism, and RNA splicing
Zhang et al.^36^	Investigate the role of mitophagy in PA invasiveness	19 PA samples (10 invasive PA, 9 non‐invasive PA); adenoma subtype not specified	LFQ LC‐Qexactive Orbitrap MS/MS	Transcriptomics, IHC, Western Blot	NCBI	GO, KEGG, WebGestalt, Metascape, STRING, GeneCards	Tumor invasiveness.	833 DEPs identified from samples of invasive and noninvasive pituitary adenomas	Mitophagy identified as the most significantly altered pathway in invasive adenomas; *HSPD1* downregulation contributes to invasive behavior.

^a^ Data‐dependent acquisition (DDA) was used unless otherwise stated.

### 3.1 Proteomic differences between NFPAs and the Normal pituitary glands

Fifteen studies analyzed NFPAs relative to normal pituitary tissue to explore molecular mechanisms underlying tumorigenesis. The analyses included differences in protein expression and PTMs involved in various metabolic pathways.^8^, ^9^, ^11^, ^13^, ^19^, ^20^, ^21^, ^24^, ^25^, ^27^, ^28^, ^30^, ^31^, ^32^, ^34^ Most studies included small exploratory cohorts, with one exception analyzing 200 pituitary adenomas (100 NFPAs) and an independent validation cohort of 750 patients.^32^ Importantly, most studies did not classify NFPA subtypes according to the current WHO transcription factor‐based system, thereby limiting comparability across cohorts.

Early, primarily exploratory studies with relatively small cohort sizes identified differentially expressed proteins (DEPs) in NFPAs relative to normal pituitary tissue. Zhan et al.^8^ reported significant downregulation of secretagogin at both protein and mRNA levels, whereas Moreno et al.^9^ identified 21 upregulated and 29 downregulated proteins and implicated dysregulation of Wnt and Notch signaling in tumorigenesis. In a subsequent study with similarly limited cohort sizes, Zhan et al.^13^ showed that NFPA subtypes exhibit distinct proteomic signatures, with MAPK signaling, oxidative stress, mitochondrial dysfunction, and cell cycle dysregulation differing between subtypes, as well as relative to the normal pituitary gland.

A phosphoproteomic analysis based on a small exploratory cohort, which mapped phosphorylation‐dependent regulation of protein activity, subsequently expanded on these findings. Li et al.^27^ reported 595 differentially phosphorylated proteins, highlighting alterations in the spliceosome, RNA transport, and the *Proteoglycans in cancer* signaling pathway. In parallel, several small studies consistently reported spliceosome dysregulation, including altered expression of spliceosome components, such as SRSF1, U2AF1, and RBM42.^25^ Spliceosome pathways were similarly implicated, specifically through upregulation of SRC and AKT1 as potential therapeutic targets.^19^

A multi‐omics study of pituitary adenomas identified metabolic alterations in NFPAs,^20^ with the authors observing a general downregulation of glucose metabolism and glycolysis across subtypes of both functioning and nonfunctioning adenomas. As part of this broader multi‐omics characterization, they also identified subtype‐specific metabolic patterns that allowed classification into three metabolic clusters. The metabolomes of oncocytoma, gonadotrophic adenomas, and null cell adenomas appeared closely related and differed from those of the other two clusters.

Zhang et al.^32^ provided broader insights into NFPA heterogeneity by combining genomic, transcriptomic, proteomic, and phosphoproteomic data from 200 adenomas (100 NFPAs). They identified distinct molecular subtypes, with NFPAs clustering into SF‐1/NULL‐, silent PIT1‐, and silent TPIT‐enriched groups, each showing unique pathway profiles. Hypoxia and VEGF signaling pathways were uniquely upregulated in both SF1 lineage and NULL tumors, suggesting that angiogenesis inhibitors targeting VEGFR2 may represent potential therapeutic strategies for these subgroups. Although genetic alterations, such as those in *GNAS*, were also observed, these primarily involved PIT1‐lineage tumors rather than NFPAs.

### 3.2 Proteomic patterns associated with tumor progression

Two studies investigating proteomic differences linked to tumor progression in NFPAs evaluated the recurrence and regrowth of residual tumors.^29^, ^35^ By analyzing 20 patients with mixed NFPA subtypes and a mean follow‐up of 10 years, Rai et al.^29^ defined progression as radiological tumor regrowth comprising a ≥20% increase in volume or ≥2‐mm growth in any dimension. Using phosphoproteomics, they identified β‐catenin phosphorylation at Ser552 (pSer552) as a recurrence marker that correlated with shorter recurrence‐free survival, thereby implicating activated Wnt signaling in tumor progression. These findings were validated by immunohistochemistry in an independent cohort of 200 NFPA patients, including 44 demonstrating recurrence during long‐term follow‐up. Although receiver operating characteristic analysis confirmed the predictive potential of β‐catenin (pSer552) as a biomarker, moderate discriminatory power ultimately limited its clinical utility.

Hallén et al.^35^ examined 46 patients in a retrospective cohort of progressive versus stable NFPA (gonadotrophic tumor subtype) with a mean follow‐up of approximately 10 years. Progression was defined as regrowth of postoperative residual tumors requiring reintervention (surgery or radiotherapy) and the study identified 550 DEPs enriched in pathways related to translation, RNA splicing, and energy metabolism. Specifically, upregulated expression of Sm‐D1 (*SNRPD1*) and SRSF10 implicated dysregulated RNA processing, whereas upregulated SWAP‐70 (*SWAP70*) expression suggested alterations in cytoskeletal dynamics and signaling related to cell migration. These findings have not been validated in an independent cohort, limiting the generalizability of the results.

These studies collectively suggest roles for RNA processing and phosphorylation‐mediated Wnt signaling activation in NFPA progression. However, the available evidence remains limited to findings from these two cohorts.

### 3.3 Proteomic differences between invasive and noninvasive NFPAs

Proteomic differences between invasive and noninvasive NFPAs have been investigated in 16 studies, the majority of which are based on small exploratory cohorts.^12^, ^15^, ^16^, ^17^, ^18^, ^21^, ^22^, ^23^, ^24^, ^26^, ^28^, ^29^, ^30^, ^32^, ^33^, ^36^ Invasiveness was often defined using radiological criteria and was most often according to the Knosp grading system, although some studies relied on intraoperative findings or combined radiological and histological features. NFPA subtypes were rarely stratified according to current WHO transcription factor‐based classifications, which limits comparability across cohorts.

Several studies reported that invasive NFPAs display alterations in the extracellular matrix (ECM) and cytoskeleton. Expression of the collagen subunit Collagen alpha‐1(XI) chain (*COL11A1*) was specifically identified as highly upregulated in invasive tumors,^15^ and Wang et al.^22^ reported overexpression of a broader set of collagens and laminins. Osteopontin and caldesmon were linked to bone invasion and destructive growth,^18^ with matrix metalloproteinases also implicated in invasive behavior.^15^, ^30^ A large integrative multi‐omics analysis further associated invasiveness with epithelial–mesenchymal transition (EMT) and ECM remodeling, as well as the enrichment of EMT markers.^32^ In a subsequent study from the same group, GLUT1 (*SLC2A1*) was also identified as a potential EMT‐related biomarker.^33^

Yu et al.^16^ reported decreased Chromogranin A (*CHGA*) and increased Clusterin (*CLU*) expression in invasive tumors, with these findings validated by qRT‐PCR and Western blot. The same study also identified differential expression of Ezrin, with Ezrin later validated as an invasion‐associated protein with functional relevance.^17^ Additionally, expression of HINT1 is reportedly upregulated in invasive tumors,^26^ and Heat shock protein 60, mitochondrial (*HSPD1*) was recently suggested as a marker of invasiveness linked to mitophagy.^36^

Recurrent dysregulation of PI3K–Akt, MAPK, Wnt, and IL6/JAK/STAT3 signaling has been observed across several cohorts. Feng et al.^15^ identified overactivation of IL6/JAK2/STAT3 signaling along with upregulated MMP‐9 (*MMP9*) expression in invasive tumors, whereas integrative analyses confirmed roles for PI3K–Akt, MAPK, and Wnt signaling in invasive behavior.^21^, ^24^ Other DIA and multi‐omics studies further substantiated the central role of ECM remodeling and associated signaling pathways in invasive NFPAs.^30^, ^32^, ^33^

PTM‐focused approaches, applied across cohorts of varying size and design, have provided additional insight into the mechanisms underlying NFPA invasiveness. Liu et al.^24^ integrated phosphoproteomics and transcriptomic data to identify molecular mechanisms of invasiveness. Pathway analyses revealed enrichment of signaling networks involved in cell adhesion, GTPase and kinase activity, calcium and hormone signaling, supporting phosphorylation‐mediated mechanisms of tumor invasiveness. Rai et al.^29^ reported significant phosphorylation of β‐catenin at Ser552 in recurrent and invasive tumors relative to nonaggressive tumors, linking this modification to Wnt pathway activation and aggressive clinical behavior. In an integrative multi‐omics analysis, Zhang et al.,^32^ identified EGFR phosphorylation in an invasive tumor cluster of silent TPIT‐tumors, suggesting that this phosphorylation event may be associated with tumor invasiveness. Wen et al.^28^ identified 26 acetylation‐regulated proteins overlapping with invasion‐associated, differentially expressed genes from transcriptomic data, mainly involved in metabolism‐related pathways.

### 3.4 Shared pathways implicated in NFPA behavior

**FIGURE 3:**
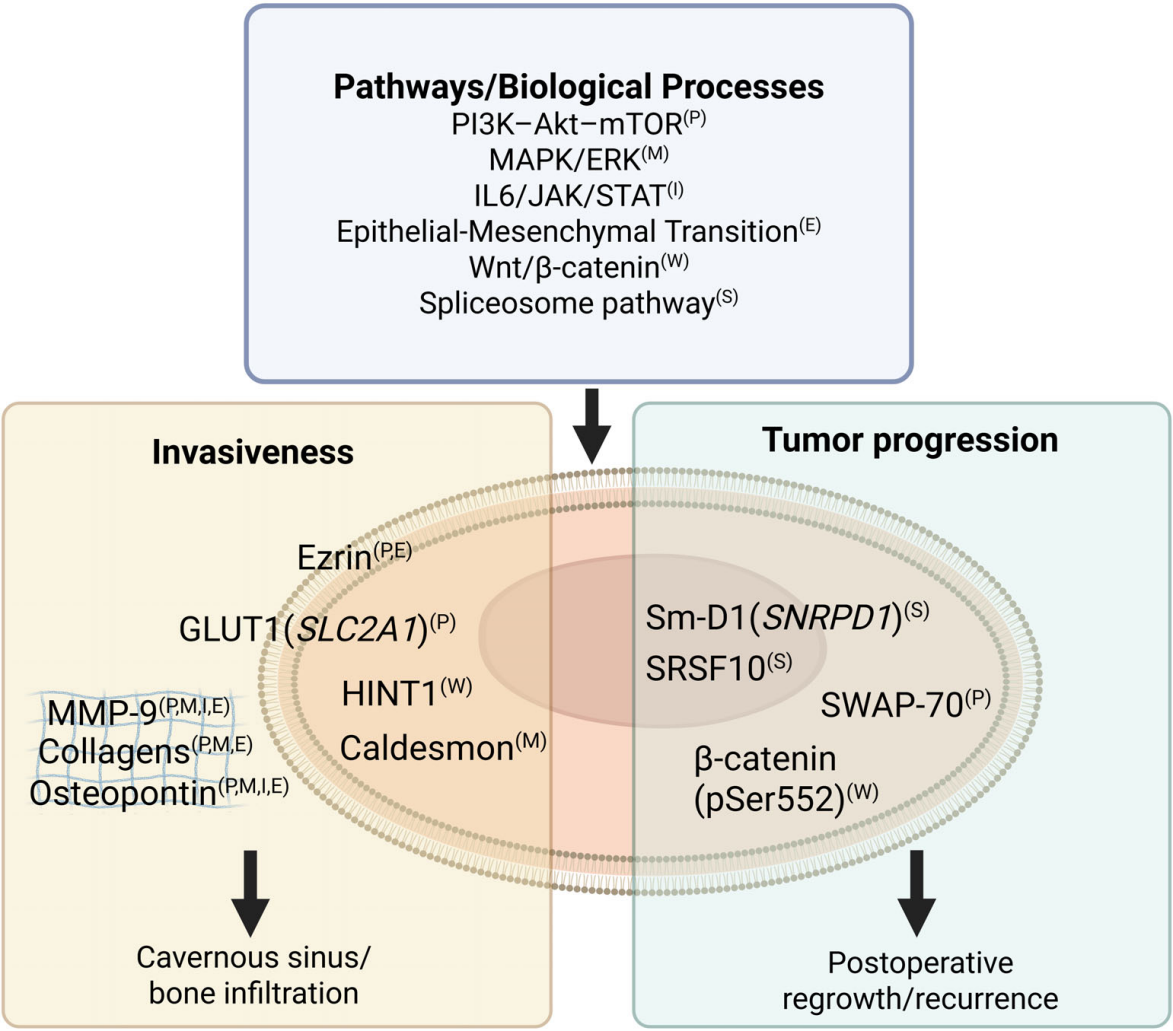
Proteomic findings in NFPAs suggesting associations with invasiveness and progression. Molecules are presented according to the nomenclature used in the original studies. When a protein name was reported, it is shown unchanged. When only a gene name was provided, the corresponding protein name is shown first, followed by the italicized gene symbol in parentheses; PTMs are shown in regular font within parantheses. Proteins are positioned according to their predominant subcellular localization, not based on experimentally verified localization data. Superscript letters denote putative pathway or biological process connections. Findings should be interpreted with caution, given the limited size and heterogeneity of the studies and the absence of independent validation.

Several studies reported similar alterations in major signaling pathways in NFPAs, including MAPK/ERK, PI3K–Akt–mTOR, Wnt/β‐catenin, and IL6/JAK/STAT3.^9^, ^12^, ^13^, ^14^, ^15^, ^21^, ^22^, ^24^, ^29^, ^31^, ^32^, ^33^ These pathways were identified across all three thematic areas, including comparisons with normal pituitary tissue and analyses of tumor progression and invasiveness. These pathways regulate fundamental processes, such as proliferation, survival, metabolism, and inflammatory responses, and likely represent core features of NFPA biology. Table 2 summarizes altered pathways and biological processes reported by at least two studies. Key pathways and proposed proteins associated with invasiveness and tumor progression are summarized in Figure 3.

**TABLE 2:** Summary of altered pathways and biological processes reported in at least two studies of nonfunctioning pituitary adenomas. All abbreviations used in this table are defined in Supplementary Table S2.

Pathway/biological process	Biological role	Examples of representative proteins (accession no., *gene name*)	Examples of observed changes in NFPAs	Possible clinical relevance	References
PI3K‐Akt–mTOR signaling pathway	Supports cell survival, growth, and metabolism.	AKT1 (P31749, *AKT1*) GSK‐3β (P49841, *GSK3B*) PRAS40 (Q96B36, *AKT1S1*)	↓GSK‐3β in NFPAs vs. pituitary gland PI3K‐Akt signaling enriched in invasive NFPAs	Possible therapeutic target for invasive tumors	Wang et al.^22^ Long et al.^21^ Liu et al.^24^ Zhang et al.^32^
MAPK/ERK signaling pathway	Regulates cell proliferation, differentiation, and apoptosis	ERK2 (P28482, *MAPK1*) BRAF (P15056, *BRAF*) MEK1 (Q02750, *MAP2K1*)	MAPK signaling reported as altered between NFPA subtypes	Potential target for subtype‐specific treatment or biomarker development	Zhan et al.^12^, ^13^ Wang et al.^14^ Long et al.^21^ Liu et al.^24^ Tanigushi‐Ponciano et al.^31^
IL6/JAK/STAT3 pathway	Mediates inflammation, immune signaling, and cell survival	IL‐6R (P08887, *IL6R*) JAK‐2 (O60674, *JAK2*) STAT3 (P40763, *STAT3*)	↑IL‐6R, JAK2, STAT3 in invasive adenomas	Possible therapeutic target for invasive tumors	Feng et al.^15^ Zhang et al.^32^ Zhang et al.^33^
Epithelial–mesenchymal transition (EMT)	Supports tissue remodeling, cell motility, and survival	TWIST1 (Q15672, *TWIST1*) Fibronectin 1 (P02751, *FN1*) Cadherin‐1 (P12830, *CDH1*)	↑EMT markers linked to increased invasiveness	Possible therapeutic target for invasive tumors.	Zhang et al.^32^ Zhang et al.^33^
Wnt/β‐catenin signaling pathway	Regulates cell fate, tissue development, and proliferation	β‐catenin (P35222, *CTNNB1*) GSK‐3β (P49841, *GSK3B*) sFRP‐1 (Q8N474, *SFRP1*)	↑β‐catenin in NFPAs vs. pituitary gland ↑phosphorylation of β‐catenin at Ser552 in recurrent and invasive NFPA	Phosphorylation of β‐catenin at Ser552 is a possible biomarker of recurrence	Moreno et al.^9^ Long et al.^21^ Rai et al.^29^
Spliceosome pathway	Regulates mRNA splicing, transcript diversity, and gene expression	SNRPD1 (P62314, *SNRPD1*) SRSF1 (Q07955, *SRSF1*) SRSF10 (O75494, *SRSF10*)	↑SNRPD1 and SRSF10 in recurrent NFPAs	Upregulated expression of spliceosome proteins may serve as a biomarker for risk of postoperative regrowth	Taniguchi‐Ponciano et al.^25^ Li et al.^27^ Hallén et al.^35^
VEGF signaling pathway	Promotes angiogenesis, vascular permeability, and cell survival	VEGFA (P15692, *VEGFA*) VEGFR2 (P35968, *KDR*)	VEGF signaling upregulated in SF1 and null cell NFPAs versus PIT1 and TPIT lineages	Angiogenesis inhibitors may serve as effective therapeutic approaches for NFPA subtypes	Wang et al.^14^ Long et al.^21^ Wang et al.^22^ Zhang et al.^32^
Focal adhesion pathway	Mediates cell adhesion, migration, and signal transduction	FAK1 (Q05397, *PTK2*) SRC (P12931, *SRC*) Ezrin (P15311, *EZR*)	↑Src in NFPAs vs. pituitary gland Focal adhesion linked to invasiveness	May serve as a potential therapeutic target	Chen et al.^17^ Cheng et al.^19^ Long et al.^21^ Wang et al.^22^

## 4 DISCUSSION

This scoping review mapped the literature on proteomic analyses of NFPAs, focusing on their identification of biomarkers and dysregulated pathways linked to tumor behavior. The findings reveal a complex and heterogeneous molecular landscape, where evaluation is complicated by substantial variability in study design, patient cohorts, and proteomic methodologies, as well as the rarity of using NFPA subtype stratification according to the current WHO system. Despite these challenges, advances in proteomic techniques have provided valuable insights into tumor biology and offered hypotheses for future validation and potential clinical translation.

### 4.1 Proteomic differences between NFPAs and normal pituitary glands

Proteomic analyses comparing NFPAs with normal pituitary glands revealed differences in protein expression, suggesting potential mechanisms underlying tumorigenesis. Early proteomic studies, despite technological limitations, identified molecular differences between NFPAs and normal pituitary tissue, highlighting alterations in Wnt and Notch signaling, as well as levels of oxidative stress and mitochondrial dysfunction. Subsequent advances in high‐resolution MS and phosphoproteomics revealed large numbers of DEPs and PTMs. Metabolic reprogramming, including altered glucose metabolism, is suggested as a contributor to NFPA pathogenesis.

The differences detected between NFPAs and normal pituitary tissue must be interpreted in the context of fundamental biological disparities between tissues. NFPAs are often considered monoclonal tumors primarily comprising a single population of transformed endocrine cells and with limited contributions from stromal elements, such as fibroblasts and blood vessels.^38^ By contrast, the normal pituitary gland is a highly heterogeneous organ comprising multiple endocrine and non‐endocrine cell types, each with distinct proteomic profiles. This intrinsic cellular diversity likely contributes to the observed proteomic differences and may confound identification of tumor‐specific alterations, underscoring the need for approaches capable of distinguishing cell‐type‐specific proteomes. Despite these challenges, comparative proteomic studies remain valuable for identifying candidate biomarkers and pathways potentially critical in NFPA tumorigenesis.

### 4.2 Proteomic differences associated with tumor progression

The presence of residual tumor tissue postsurgery for NFPAs is a common clinical challenge, with up to 50% of patients experiencing progression from the residual lesion.^39^, ^40^ This progression is associated with increased mortality, highlighting the need for better predictive tools and improved postoperative management strategies.^3^ Despite the importance of understanding tumor progression, this review identified only two studies specifically investigating the molecular mechanisms of tumor progression.^29^, ^35^ This limited number likely reflects the methodological challenges associated with studying these slow‐growing tumors, as long‐term follow‐up is often required to detect significant volumetric changes.

Proteomic analyses have nevertheless provided early insights. Hallén et al.^35^ reported widespread dysregulation of protein translation, RNA splicing, and energy metabolism in progressive tumors, with several upregulated proteins also linked to aggressiveness in other cancers. Additionally, Rai et al.^29^ identified phosphorylation of β‐catenin at Ser552 as a potential recurrence biomarker correlated with shorter recurrence‐free survival and implicated Wnt signaling activation in NFPA progression.

These findings suggest that proteomic biomarkers may improve risk stratification and postoperative management. Reliable prognostic markers would allow earlier identification of high‐risk patients, enabling tailored surveillance and intervention strategies.

### 4.3 Proteomic markers of invasiveness

Sixteen studies (>50% of the included articles) investigated proteomic differences between invasive and noninvasive NFPAs. The large number of studies adopting this approach likely reflects the clinical importance of invasiveness as a determinant of treatment outcomes, as well as the relative ease of assessing this variable compared with progression. Invasiveness can be evaluated preoperatively using MRI and/or intraoperatively based on surgical findings. This contrasts with tumor growth, which requires long‐term follow‐up for clinically meaningful assessment.

Across studies, IL‐6R/JAK2/STAT3/MMP9 and MAPK signaling emerged as central pathways driving invasiveness through ECM remodeling and oxidative stress. Specific proteins like Ezrin, GLUT1, and heat shock protein 60, mitochondrial have been linked to invasive tumor behavior, and PTMs, including phosphorylation and acetylation, reportedly play a key role in regulating invasive phenotypes. The tumor microenvironment also appears critical, with osteopontin and caldesmon associated with ECM degradation in bone‐destructive NFPAs. These studies highlight ECM remodeling, signaling pathway dysregulation, and PTMs as recurrent features of NFPA invasiveness, although definitions of invasiveness and methodological approaches remain heterogeneous.

### 4.4 Shared pathways implicated in NFPA behavior

Several of the included studies identified common pathways dysregulated in NFPAs, reinforcing their potential role in tumor behavior. Recurrent alterations in PI3K–Akt–mTOR, MAPK/ERK, IL6/JAK/STAT3, and Wnt/β‐catenin signaling suggest that NFPA biology is driven by a network of interconnected pathways rather than isolated molecular events. MAPK/ERK hyperactivation appears linked to proliferative and invasive phenotypes, whereas increased PI3K‐Akt signaling contributes to survival, metabolic adaptation, and potential treatment resistance. Wnt/β‐catenin signaling, including elevated levels of β‐catenin phosphorylation at Ser552, is also associated with recurrence and aggressive growth. Alterations in the PI3K–Akt signaling pathway, including downstream mTOR signaling, highlight tumor adaptation under metabolic stress, and activation of IL6/JAK/STAT3 signaling suggests an inflammatory component in invasive tumors. These findings identify potential therapeutic targets and emphasize the importance of pathway cross‐talk in NFPA progression. Notably, several of the recurrently altered signaling pathways identified in this review are well known drivers across multiple tumor types; however, their consistent detection in NFPA‐focused proteomic studies suggests that they are also relevant to NFPA biology and are unlikely to represent incidental findings.

### 4.5 Advances in proteomic methodology and implications for NFPA research

Early proteomic studies relied on 2D‐GE and matrix‐assisted laser desorption/ionization mass spectrometry (MALDI‐MS). Although these techniques enabled the first global protein analyses, they were limited in reproducibility, sensitivity, and dynamic range. The introduction of LC–MS/MS along with quantitative strategies, such as chemical labelling (tandem mass tag, *TMT*; isobaric tags for relative and absolute quantitation, *iTRAQ*), and label‐free quantitative (LFQ) approaches has substantially improved both the depth and quantitative accuracy of proteomic analyses. In addition, PTM analyses provide a dynamic view of the proteome, revealing functional modifications that regulate signaling, folding, and localization, thereby offering deeper insights into disease mechanisms and therapeutic opportunities. More recently, high‐throughput, automated DIA platforms have further improved reproducibility and enabled the analysis of large‐scale cohorts. Combined with bioinformatics‐driven approaches, including machine learning, these advances now facilitate the identification of pathway dysregulation and tumor‐specific proteomic signatures that may support further patient classification and stratification.

These methodological advances have already generated clinically meaningful results in cancer research. Proteomic analyses have facilitated the discovery of diagnostic, prognostic, and therapeutic biomarkers by revealing protein alterations associated with tumor development, progression, and treatment response. Proteomics has advanced research in breast cancer, lung cancer, colorectal cancer, and hepatocellular carcinoma by helping identify unique protein profiles linked to tumor behavior and patient outcomes, as well as novel drug targets.^6^, ^41^ Similar approaches have also been applied to benign tumors, such as ovarian adnexal masses, where proteomic panels distinguish benign from malignant lesions with high accuracy.^42^ By providing insights into tumor‐specific protein networks and pathways, proteomics not only enhances our understanding of cancer biology but also drives the development of targeted therapies and precision medicine approaches. Although these methodological advances are highly relevant for NFPA research, many studies continue to rely on small, heterogeneous cohorts and older techniques, limiting their translational value.

### 4.6 Limitations and methodological challenges

Despite advances in proteomic research, several limitations remain in current MS‐based proteomic studies of NFPAs. Sample sizes were often very small, as 16 of the 30 included studies evaluated <10 NFPAs, with three analyzing only a single adenoma. Such limited cohorts reduce statistical power and the ability to detect subtle but clinically relevant differences. By contrast, Zhang et al.^32^ analyzed 200 pituitary adenomas and validated findings in 750 independent cases, demonstrating the increased robustness achievable from larger cohorts.

Another limitation of the included studies is that approximately 25% relied on older MS techniques, such as 2D‐GE and MALDI, which have several inherent drawbacks. These methods depend on gel‐based image quantification and lack the sensitivity to detect low abundant proteins, resulting in reduced proteomic depth and lower quantification accuracy compared with later gel‐free LCMS approaches. Consequently, findings from these older studies do not fully capture the complexity and dynamic range of the NFPA proteome.

The significant heterogeneity among NFPA subtypes further complicates biomarker discovery. Few studies classified tumors according to transcription factors, limiting comparability and likely contributing to inconsistencies across datasets. Larger, well‐defined cohorts are required to capture biological variability and identify protein signatures that remain consistent. Recent methodological advances permit high‐depth analyses through their ability to quantify >10,000 proteins from a single biopsy, thereby enabling more precise subtype characterization.

Another key limitation is the lack of functional validation. Many identified proteins and pathways remain untested in independent cohorts or preclinical models, rendering their biological and clinical relevance uncertain. Prospective validation in larger cohorts and functional analyses will be essential. Notably, Zhang et al.^32^ integrated proteomics with other molecular‐level analyses and validated the results in an independent cohort, providing a framework for future studies.

Methodological variations in sample preparation, MS platforms, and bioinformatics pipelines complicate cross‐study comparisons. Integrative multi‐omics approaches can help clarify the molecular mechanisms underlying tumor behavior. Only two studies reported receiver operating characteristic analysis for potential biomarkers, with both reporting wide confidence intervals. These findings highlight that the prognostic value of reported biomarker panels remains largely untested.

### 4.7 Future directions

Future proteomic research on NFPAs should move beyond small, retrospective studies by establishing large, prospective cohorts with standardized imaging, clinical characterization, and long‐term follow‐up. However, assembling large NFPA cohorts is particularly challenging because meaningful assessment of progression requires prolonged follow‐up in these slow‐growing tumors. Consistent application of the current WHO transcription factor‐based classification will be essential for comparability across studies. Additionally, retrospective analyses remain highly valuable, particularly when using widely available formalin‐fixed paraffin‐embedded (FFPE) material. Although recently frozen tissue provides the greatest proteomic depth, advances in FFPE‐based protocols now allow robust protein quantification and enable retrospective studies in large, well‐annotated cohorts.

To ensure reproducible results, key methodological factors must be addressed. Variability beyond laboratory control should be considered, as study success depends on well‐defined, balanced cohorts with standardized sample collection and processing. Confounding and batch effects have compromised many clinical proteomic studies, highlighting the need for careful design and documentation. High variability, heterogeneity, small effect sizes, and limited sample numbers will also severely reduce statistical power and the ability to identify DEPs.

In addition to developments in data‐independent acquisition mass spectrometry (DIA‐MS), complementary approaches involving single‐cell and spatial proteomics, as well as targeted analysis of PTMs, including phosphorylation, glycosylation, acetylation, and ubiquitination, can expand biological resolution. Moreover, the emerging concept of a molecular twin offers a less technically demanding and more scalable approach. A molecular twin is a computational replica of a patient that integrates data across multiple omics layers, including genomics, transcriptomics, proteomics, metabolomics, together with clinical information. By modeling these interconnected molecular networks, molecular twins can simulate disease progression, predict treatment responses, and guide personalized therapeutic strategies. This approach leverages artificial intelligence and machine learning to dynamically update the molecular model as new omics or clinical data become available, providing a powerful tool for precision medicine and data‐driven patient stratification.

In conclusion, integration of proteomics with other ‐omics and clinical data combined with rigorous validation in independent cohorts will be critical to translating candidate biomarkers into clinically meaningful diagnostic, prognostic, and therapeutic tools.

## AUTHOR CONTRIBUTIONS

**Thomas Skoglund:** Conceptualization, investigation, data curation, writing – original draft, writing – review and editing. **Linus Köster:** investigation, data curation, writing – review and editing. **Annika Thorsell:** Resources, writing – review and editing. **Oskar Ragnarsson:** Resources, writing – review and editing. **Gudmundur Johannsson:** Funding acquisition, resources, writing – review and editing. **Tobias Hallén:** Conceptualization, investigation, data curation, writing – original draft, writing – review and editing. All authors have read and approved the final version of the manuscript.

## CONFLICT OF INTEREST STATEMENT

G.J. has served as a consultant for Crinetics, Novo Nordisk, and AstraZeneca, and has received lecture fees from Novo Nordisk and Pharmanovia. All other authors declare no conflicts of interest.

## Supporting information


**Table S1.** Literature search strategy.

**Table S2.** List of abbreviations used in the manuscript.

## Data Availability

The data that support the findings of this study are available from the corresponding author upon reasonable request.
