# Supplementary material for: Proteomic insights into the invasiveness and tumor progression of non‐functioning pituitary adenomas: A scoping review
Source: J Neuroendocrinol. 2026 Mar 7;38(3):e70148. doi: 10.1111/jne.70148 (PMC12967709; doi:10.1111/jne.70148)
Supplement: Supplementary file 1 — Table S1. Literature search strategy. [file JNE-38-e70148-s001.pdf]

Supplementary Table S1. Literature search strategy

Systematic searches were performed in PubMed, Scopus, and the Cochrane Library to identify studies applying mass spectrometry–based proteomics to non-functioning pituitary adenomas (NFPAs).

Searches were limited to English-language publications from the year 2000 onward. The search was conducted at the Central Clinical Library, Sahlgrenska University Hospital, Gothenburg, Sweden, on November 21, 2024, and updated on August 29, 2025.

Search blocks (PubMed syntax):

Block 1 – Proteomics

proteom*[tw] OR phosphoproteom*[tw] OR proteogenomic*[tw] OR mass spectrom*[tw] OR protein analysis[tw] OR protein quanti*[tw] OR relative quanti*[tw] OR absolute quanti*[tw] OR protein expression[tw] OR protein profil*[tw] OR protein mapping[tw] OR expressed protein*[tw] OR tandem mass tags[tw] OR TMT[tw] OR label-free LC-MS[tw] OR label-free quanti*[tw] OR LFQ[tw] OR data-independent acquisition[tw] OR DIA[tw] OR SRM[tw] OR selected reaction monitoring[tw] OR SILAC[tw] OR stable isotope labeling by amino acids in cell culture[tw] OR PMF[tw] OR peptide mass fingerprinting[tw] OR iTRAQ[tw] OR isobaric tags for relative and absolute quanti*[tw] OR immuno-LCM with 2D-nanoLC/MS[tw] OR MDLC[tw] OR multidimensional liquid chromatograph*[tw] OR SELDI[tw] OR surface-enhanced laser desorption/ionization[tw] OR LC-MS/MS[tw] OR LC-MS[tw] OR LCMS[tw] OR chromatography-mass spectrometry[tw] OR MRM[tw] OR multiple reaction monitor*[tw] OR PRM[tw] OR parallel reaction monitor*[tw] OR protein biomarker*[tw] OR Proteomics[MeSH] OR Proteome[MeSH] OR Mass Spectrometry[MeSH]

Block 2 – Pituitary tumors

Pituitary Neoplasms[MeSH] OR ((pituitary[tw] OR hypophys*[tw]) AND (adenoma*[tw] OR tumor*[tw] OR tumour*[tw] OR carcinoma*[tw] OR neoplasm*[tw] OR cancer*[tw])) OR NF-PitNET[tw] OR NFPitNET[tw] OR PitNET[tw] OR NFPA[tw] OR NFPAs[tw]

Search syntax notes:

[tw] = text word (searches title, abstract, and other relevant fields)

[MeSH] = Medical Subject Heading (exploded term)

*= truncation symbol for alternate endings

Combined search:

(Block 1) AND (Block 2)
