# Supplementary material for: Proteomic insights into the invasiveness and tumor progression of non‐functioning pituitary adenomas: A scoping review
Source: J Neuroendocrinol. 2026 Mar 7;38(3):e70148. doi: 10.1111/jne.70148 (PMC12967709; doi:10.1111/jne.70148)
Supplement: Supplementary file 2 — Table S2. List of abbreviations used in the manuscript. [file JNE-38-e70148-s002.pdf]

Supplementary Table S2. List of abbreviations used in the manuscript.

2D-GE	Two-Dimensional Gel Electrophoresis.
4D	fourDimensional
ACTH	Adrenocorticotropic Hormone
CDK	Cyclin-Dependent Kinase
DAVID	Database for Annotation, Visualization and Integrated Discovery
DDA	Data-Dependent Acquisition
DEP	Differentially Expressed Proteins
DIA	Data-Independent Acquisition
DPP	Differentially Phosphorylated Proteins
ECM	Extracellular Matrix
EMT	Epithelial–Mesenchymal Transition
EXPASy	Expert Protein Analysis System
FCA	Functioning Adenoma
FSH	Follicle-Stimulating Hormone
FunRich	Functional Enrichment analysis tool
GEO	Gene Expression Omnibus
GH	Growth Hormone
GO	Gene Ontology
GSEA	Gene Set Enrichment Analysis
HPLC-MS/MS	High Performance Liquid Chromatography-Tandem Mass Spectrometry
IPA	Ingenuity Pathway Analysis
IPAKB	Ingenuity Pathways Analysis Knowledge Base
iTRAQ	Isobaric Tags for Relative and Absolute Quantitation
KEEG	Kyoto Encyclopedia of Genes and Genomes
KOBAS	KEGG Orthology Based Annotation System
LC-ESI-qTOF	Liquid Separation-Electrospray Ionization-Quadrupole Time-of-Flight
LS-ESI-QIT	Liquid Separation-Electrospray Ionization-Quadrupole Ion Trap
MALDI	Matrix-Assisted Laser Desorption/Ionization
MALDI-LTQ	MALDI-Linear Trap Quadrupole
MALDI-TOF	MALDI - Time of Flight
MALDI-TOF PMF	MALDI-TOF-Peptide Mass Fingerprinting
mRNA	messenger RiboNucleic Acid
MS-FIT	Mass Spectrometry – Fast Identification Tool
MS/MS	Mass Spectrometry/Mass Spectrometry (Tandem Mass Spectrometry)
N/A	Not Applicable
nanoLC-MS/MS	Nano Liquid Chromatography-Tandem Mass Spectrometry
NCBI	National Center for Biotechnology Information
NCBInr	NCBI non-redundant protein sequence database
NFPA	Non-Functioning Pituitary Adenoma

NR5A1	Nuclear Receptor Subfamily 5 Group A Member 1
NTAC	NitroTyrosine Affinity Column
PA	Pituitary Adenoma
PANTHER	Protein ANalysis THrough Evolutionary Relationships
PCR	Polymerase Chain Reaction
PIT-1	Pituitary-Specific Positive Transcription Factor 1
POU1F1	Pit-1Oct-1Unc-86 Class 1 Homeobox 1
PRL	Prolactinoma
PTM	Post-Translational Modification
RefSeq	Reference Sequence Database
RT-PCR	Reverse Transcription Polymerase Chain Reaction
SCA	Silent Corticotroph Adenoma
SEQUEST HT	SEQUEST High Throughput
SF-1	Steroidogenic Factor 1
STRING	Search Tool for the Retrieval of Interacting Genes/Proteins
SWISS-PROT	Swiss-Prot Protein Sequence Database
TBX19	T-box transcription factor 19
TiO2	titanium dioxide
TMT	Tandem Mass Tag
TPIT	T-box Pituitary Transcription Factor
Tr-EMBL	Translated-European Molecular Biology Laboratory
